# Supplementary material for: Bioassay-Guided Isolation of Iridoid Glucosides from Stenaria nigricans, Their Biting Deterrence against Aedes aegypti (Diptera: Culicidae), and Repellency Assessment against Imported Fire Ants (Hymenoptera: Formicidae)
Source: Molecules. 2022 Oct 19;27(20):7053. doi: 10.3390/molecules27207053 (PMC9611780; doi:10.3390/molecules27207053)
Supplement: Supplementary file 1 [file molecules-27-07053-s001.zip › molecules-1965038-supplementary.pdf]

Supplementary materials

Bioassay-Guided Isolation of Iridoid Glucosides from *Stenaria nigricans*, Their Biting Deterrence Against *Aedes aegypti* (Diptera: Culicidae) and Repellency Assessment Against Imported Fire Ants (Hymenoptera: Formicidae)

Fazila Zulfiqar [1], Abbas Ali [1], Zulfiqar Ali [1] and Ikhlas A. Khan [1,2,*]

[1] National Center for Natural Products Research, School of Pharmacy, The University of Mississippi, Oxford, MS 38677, USA

[2] Department of BioMolecular Sciences, Division of Pharmacognosy, School of Pharmacy, The University of Mississippi, Oxford, MS 38677, USA

* Correspondence: ikhan@olemiss.edu; Tel.: +1-(662)-915-1090.

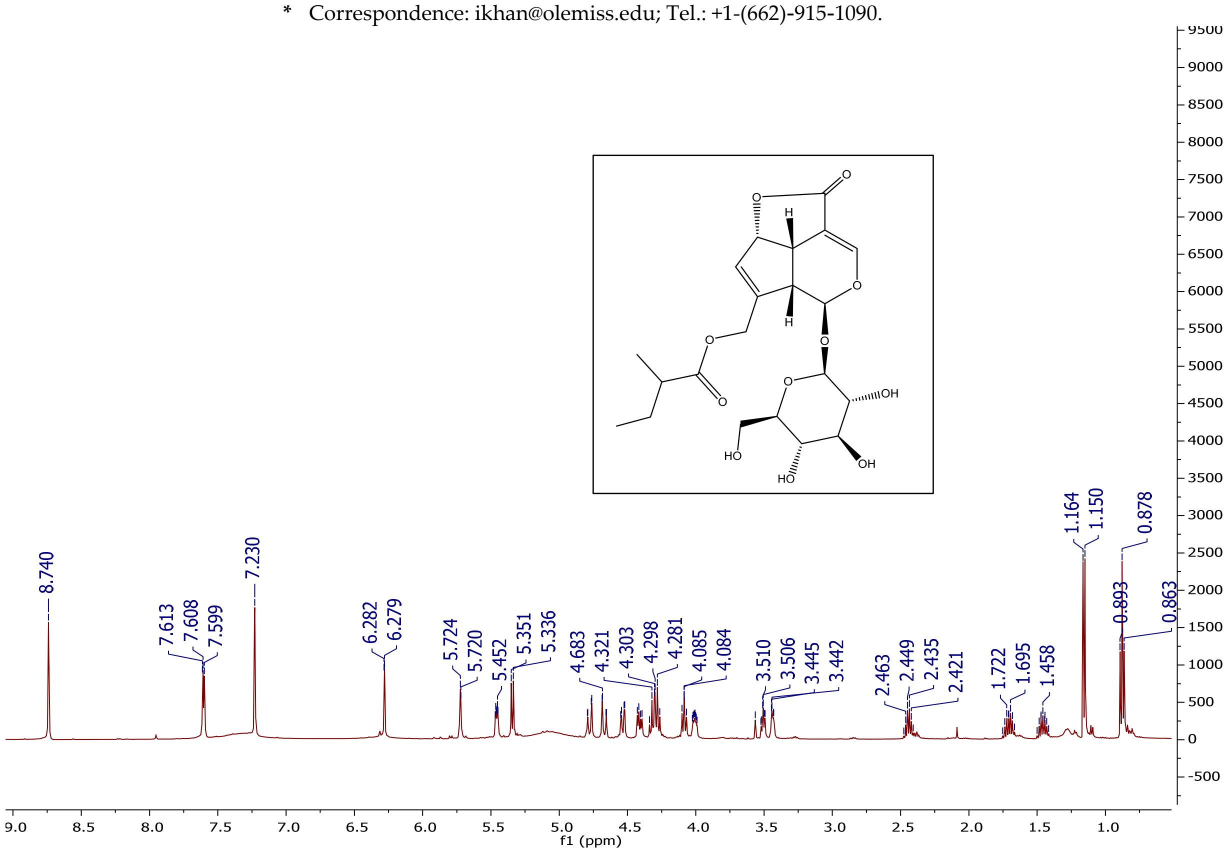

Figure S1. ^{1}HNMR spectrum of **1** at 500 MHz in pyridine-*d*5.

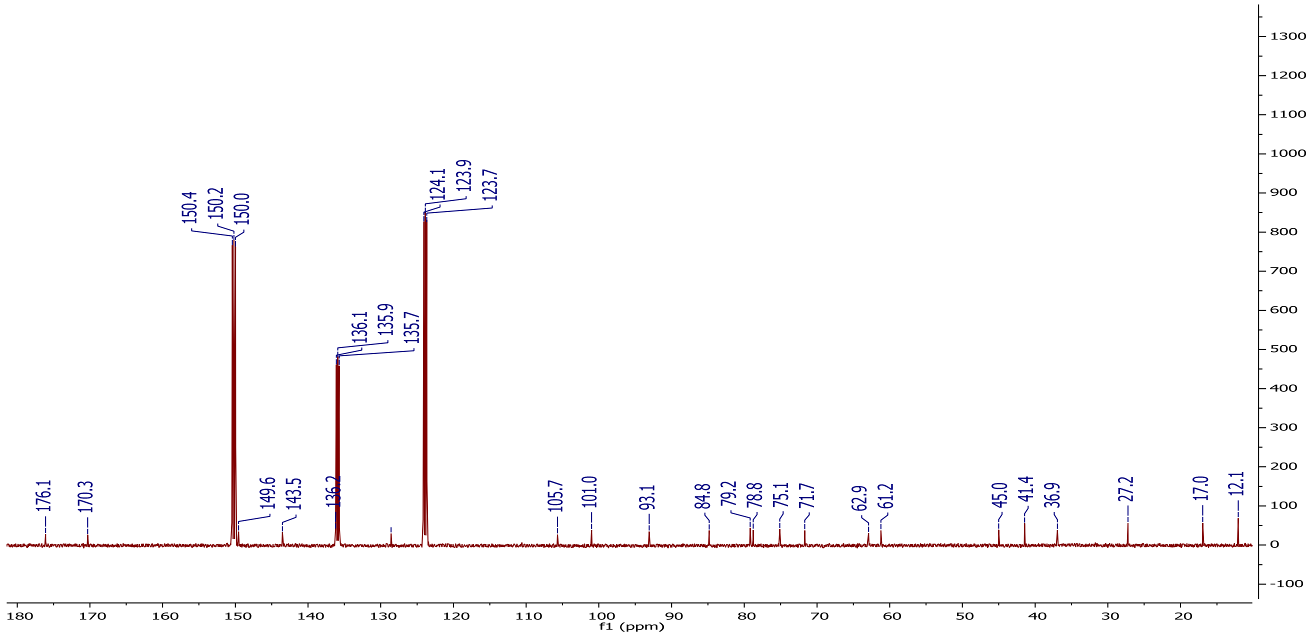

Figure S2. ^{13}CNMR spectrum of **1** at 125 MHz in pyridine-*d*5.

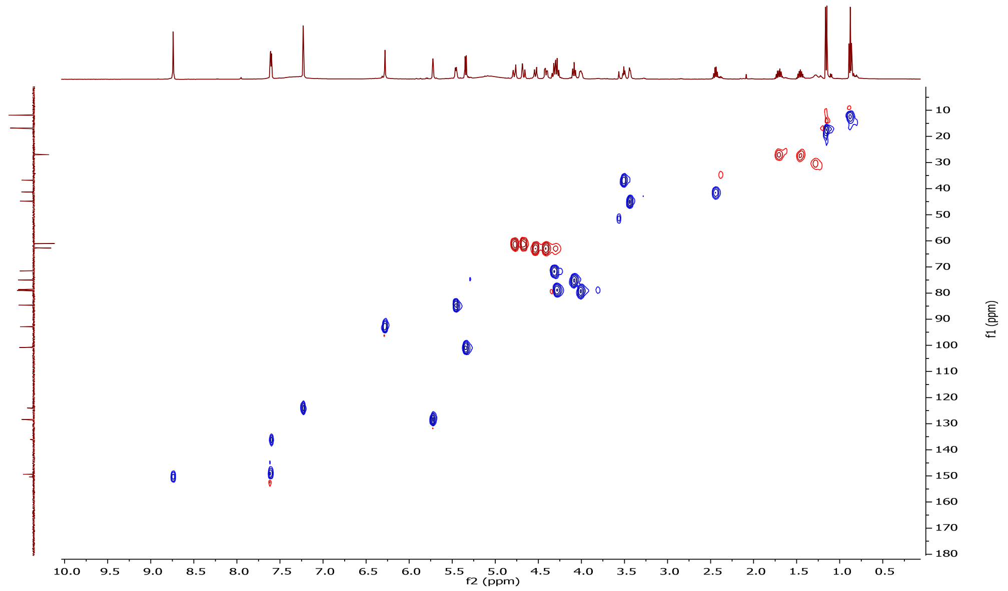

Figure S3. HSQC spectrum of **1** in pyridine-*d*5.

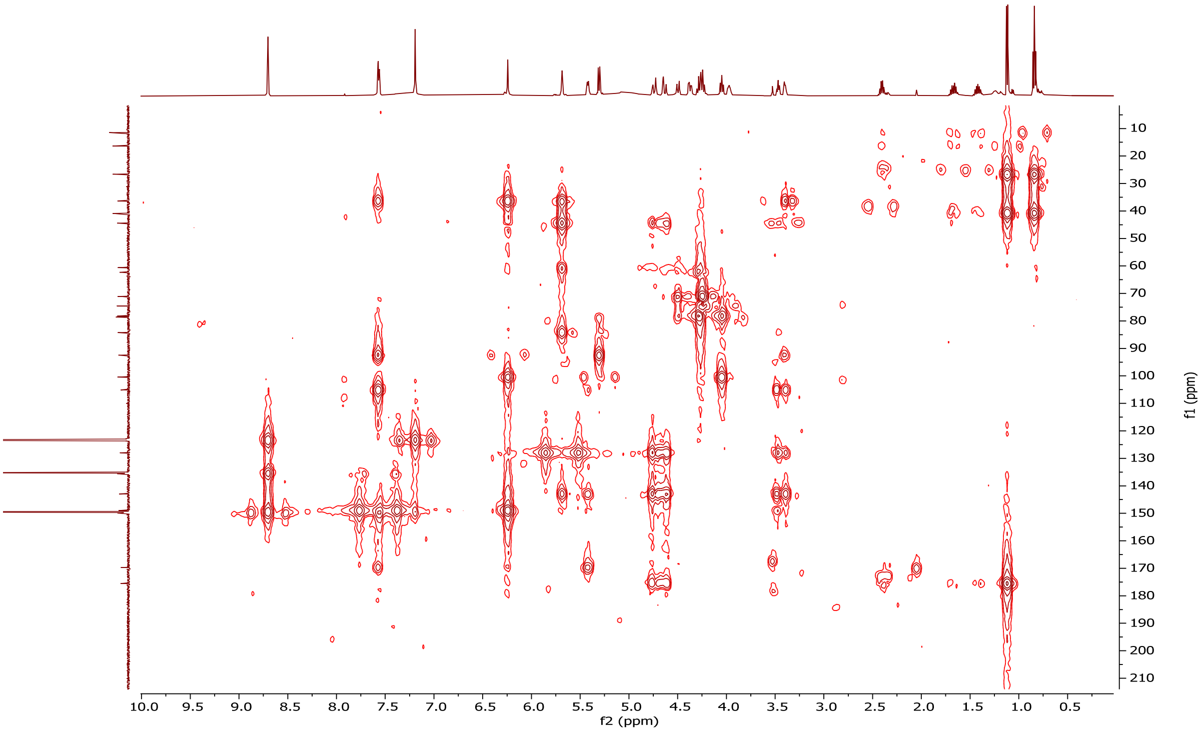

Figure S4. HMBC spectrum of **1** in pyridine-*d*5.

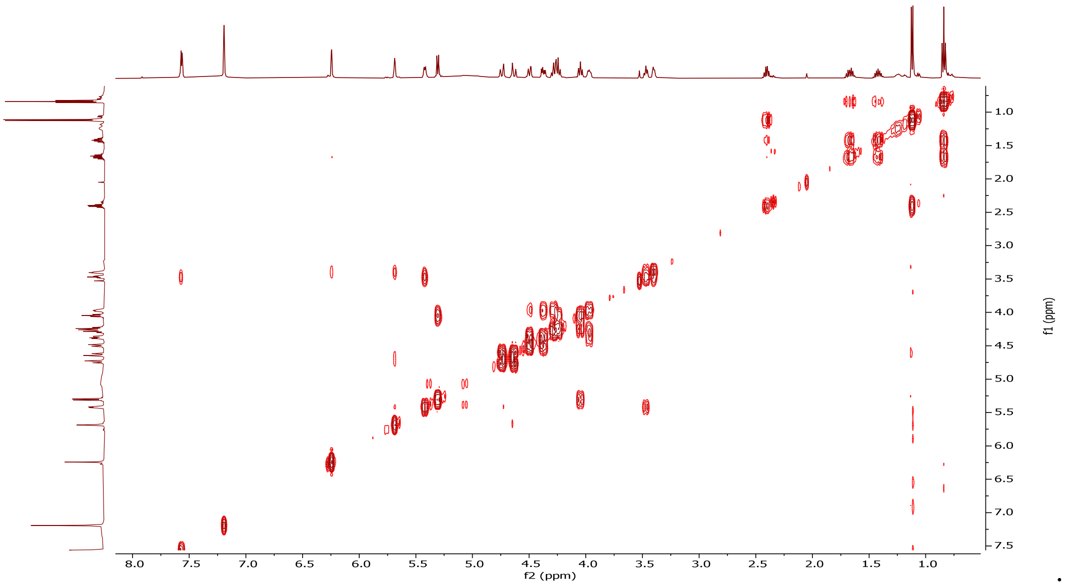

Figure S5. COSY spectrum of **1** in pyridine-*d*5.

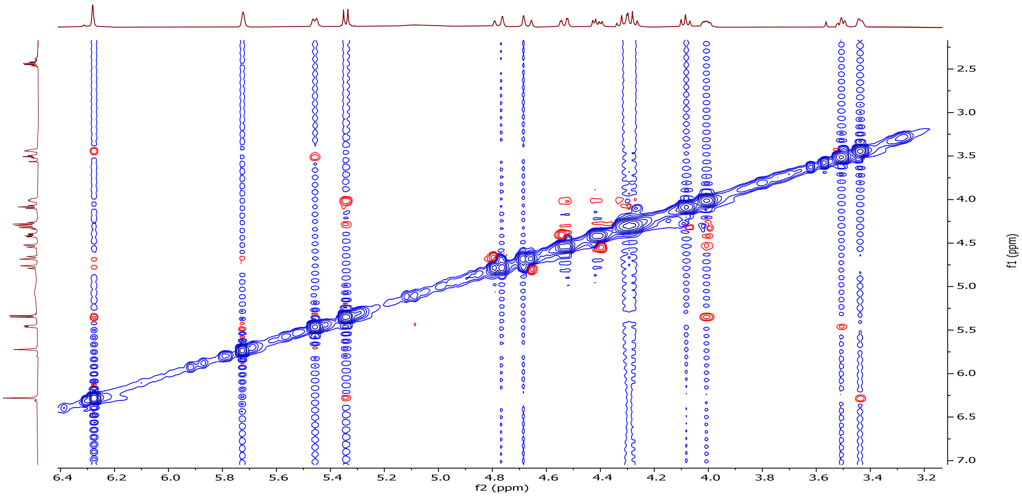

Figure S6. NOESY spectrum of **1** in pyridine-*d*5.

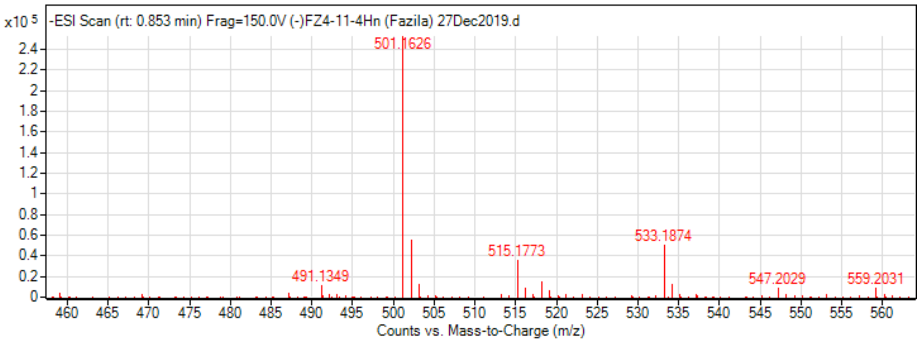

Figure S7. HRESIMS (negative ionization mode) of **1.**

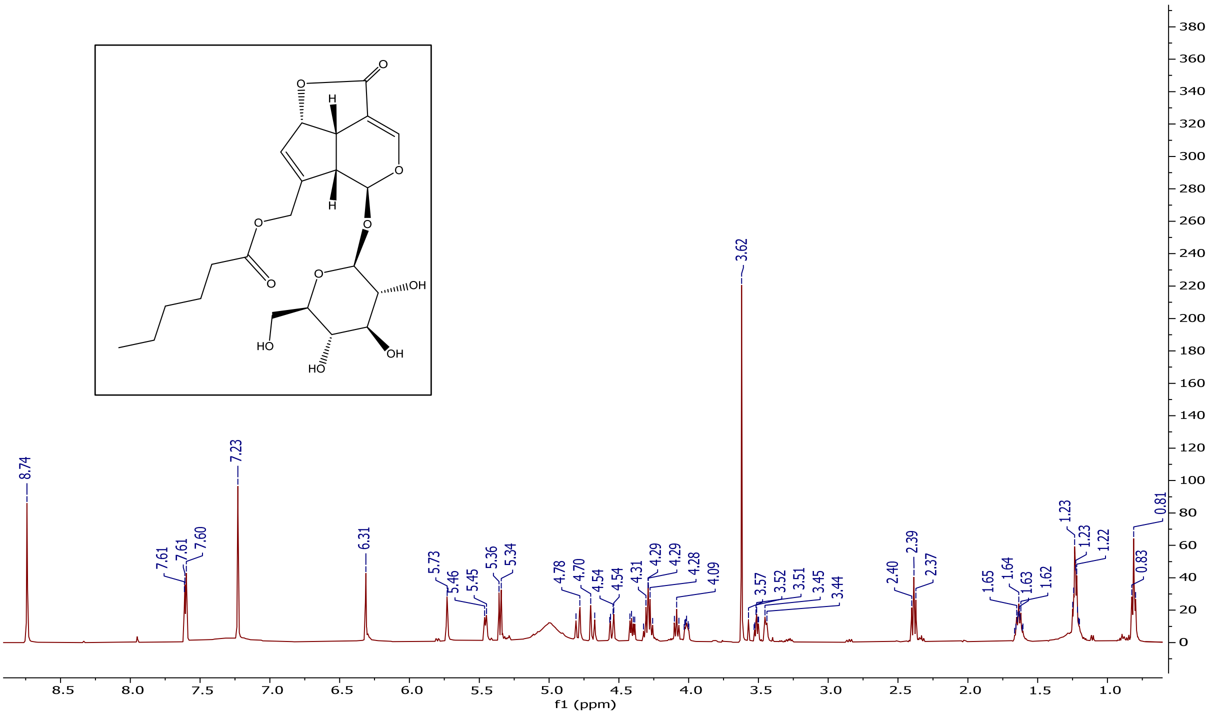

Figure S8. ^{1}HNMR spectrum of **2** at 500 MHz in pyridine-*d*5.

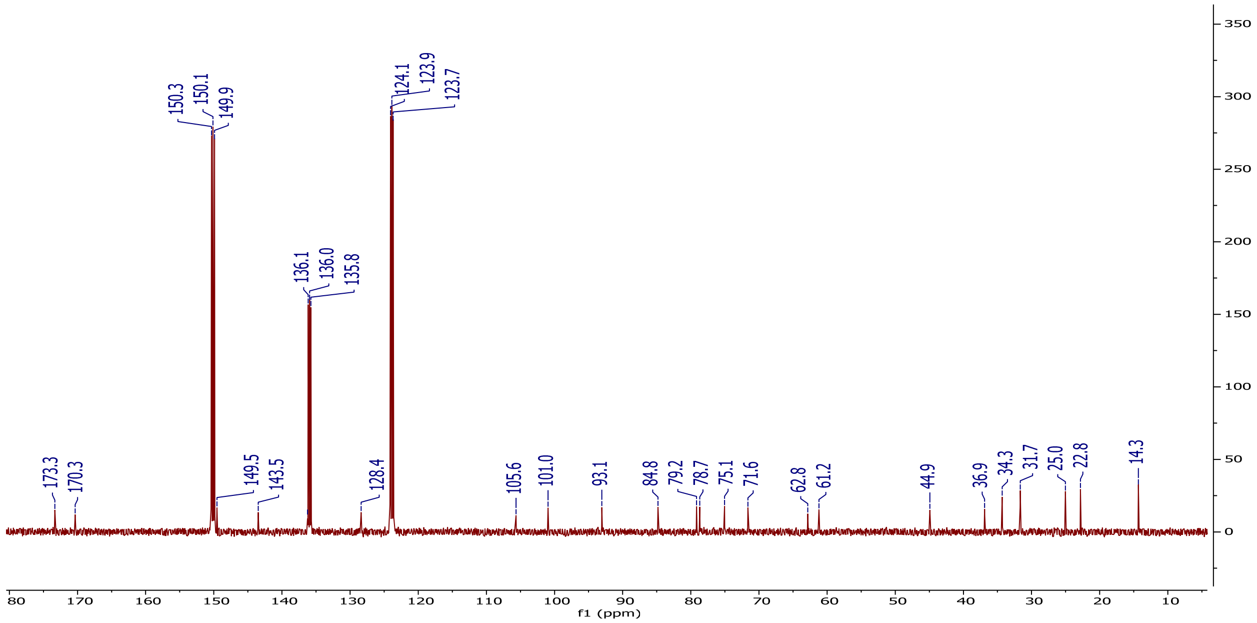

Figure S9. ^{13}CNMR spectrum of **2** at 125 MHz in pyridine-*d*5.

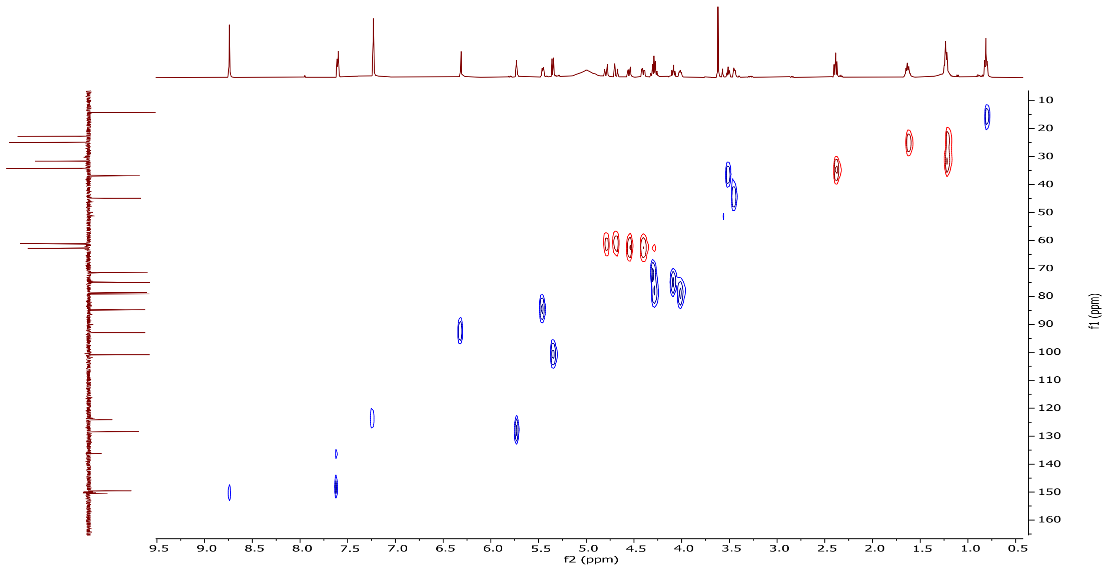

Figure S10. HSQC spectrum of **2** at in pyridine-*d*5.

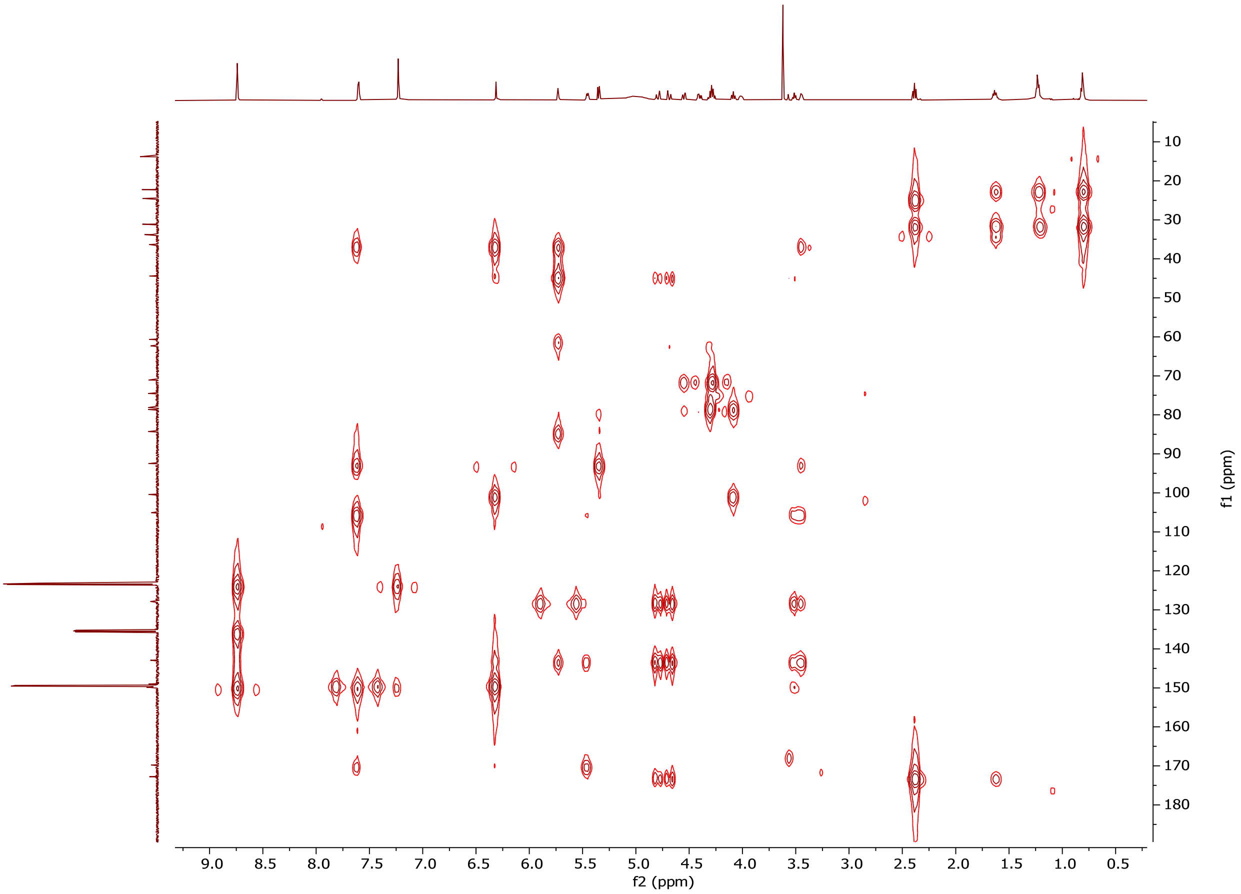

Figure S11. HMBC spectrum of **2** in pyridine-d_5.

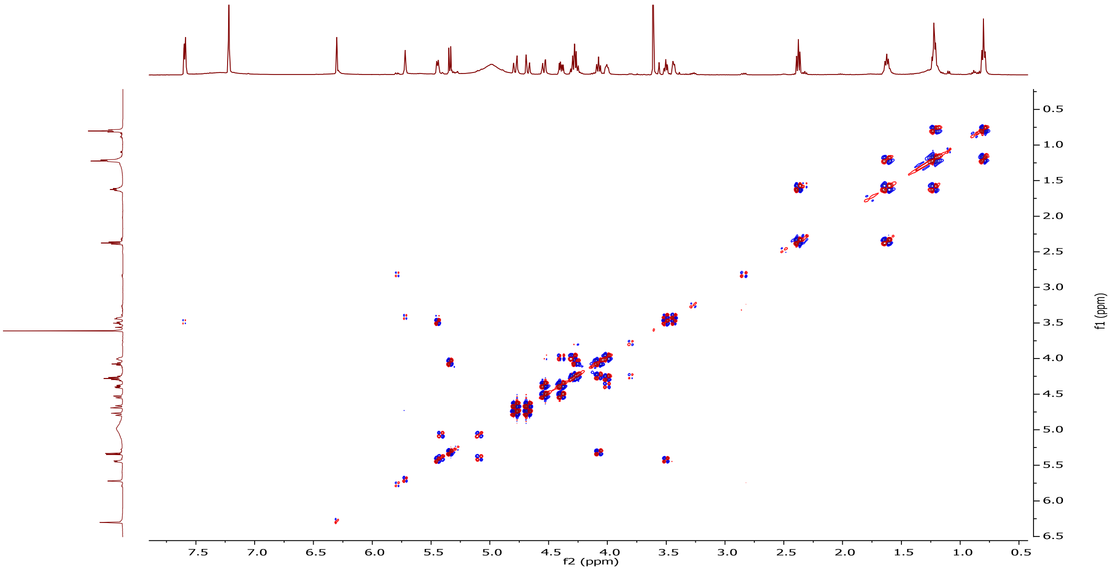

Figure S12. COSY spectrum of **2** in pyridine-d_5.

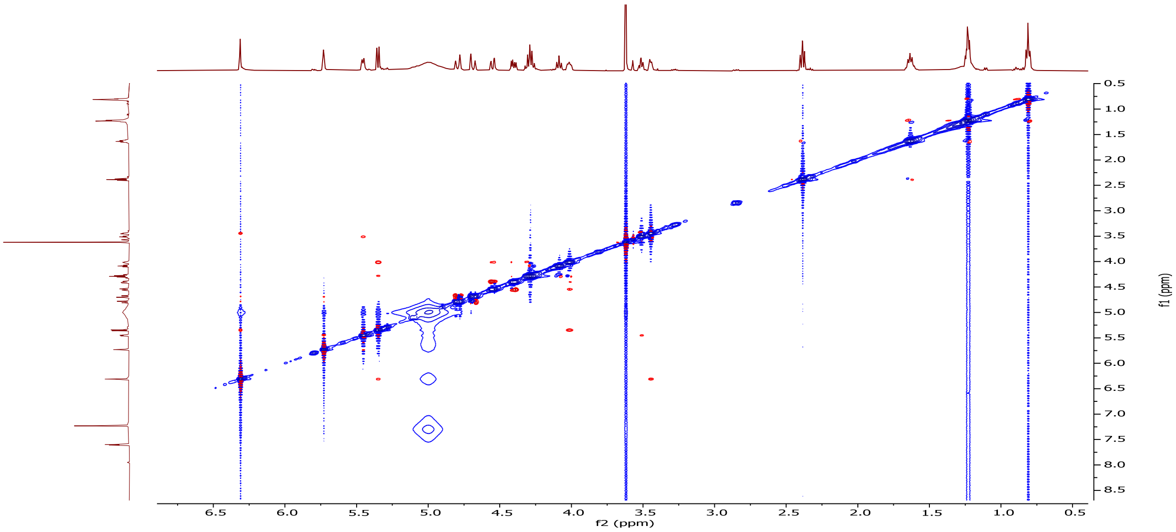

Figure S13. NOESY spectrum of **2** in pyridine-*d*5.

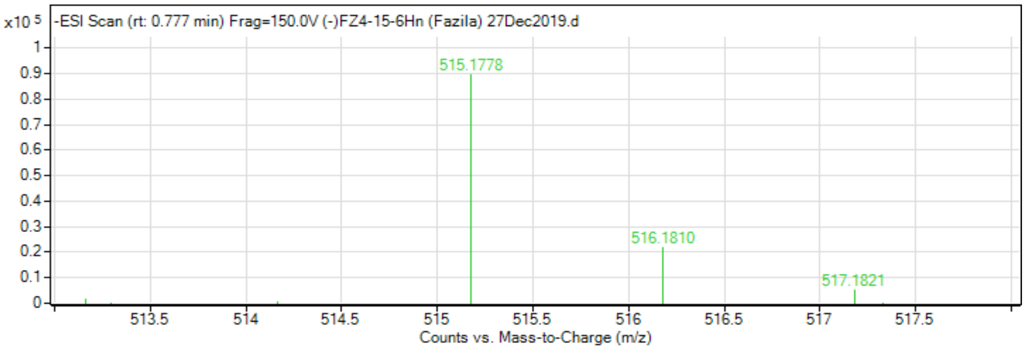

Figure S14. HRESIMS of **2**.

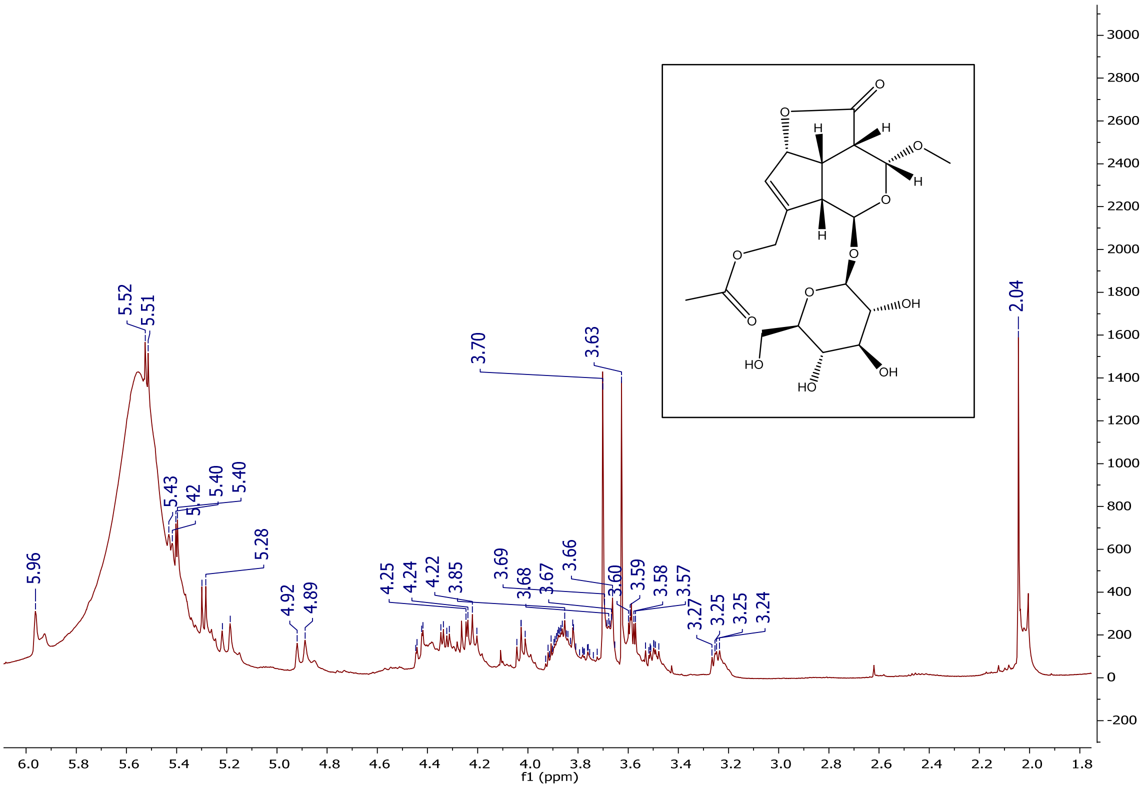

Figure S15. ^{1}HNMR spectrum of **3** at 500 MHz in pyridine-*d*5.

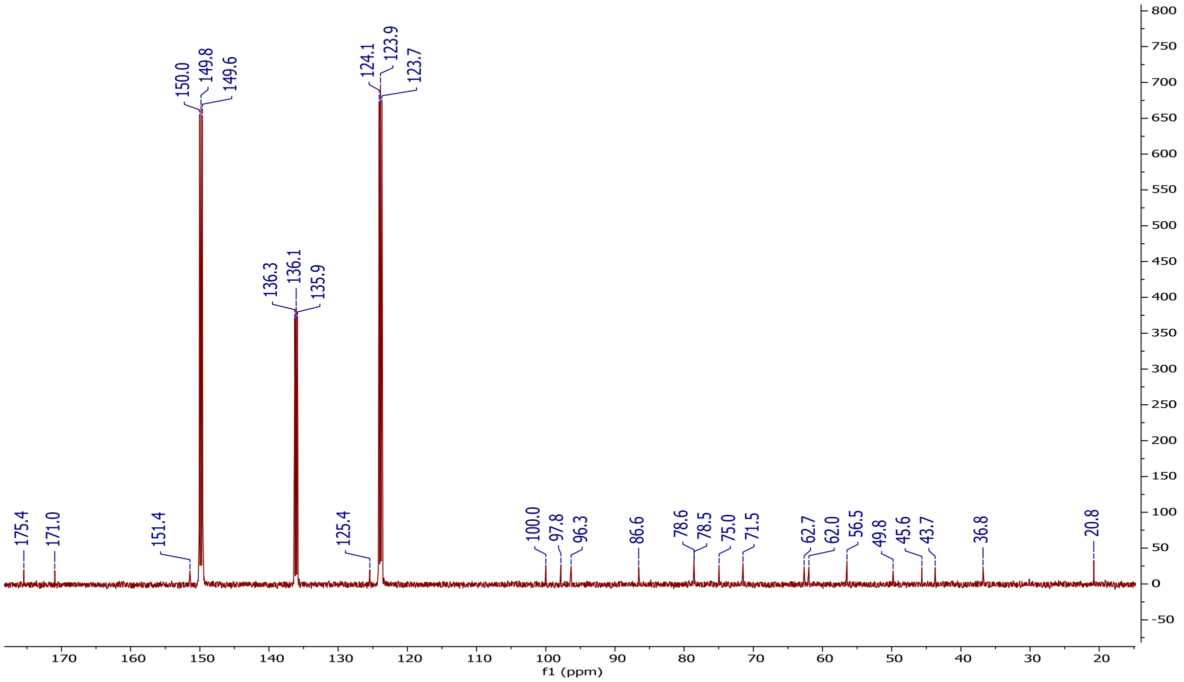

Figure S16. ^{13}CNMR spectrum of **3** at 125 MHz in pyridine-*d*5.

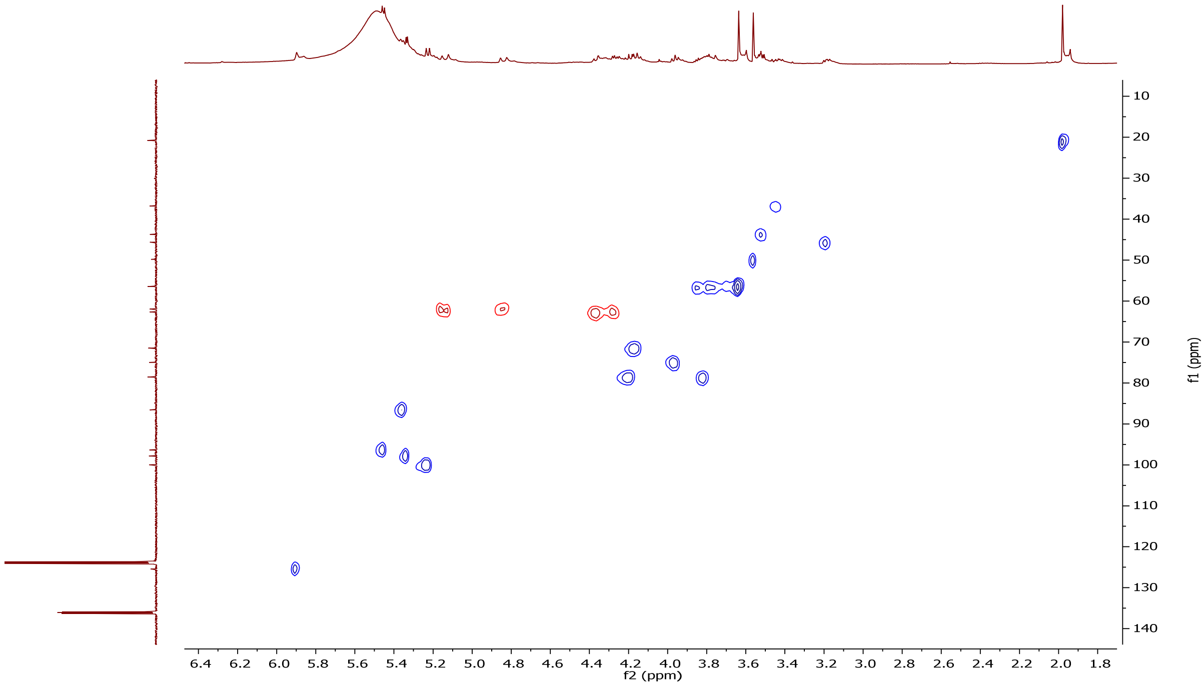

Figure S17. HSQC spectrum of **3** in pyridine-*d*5.

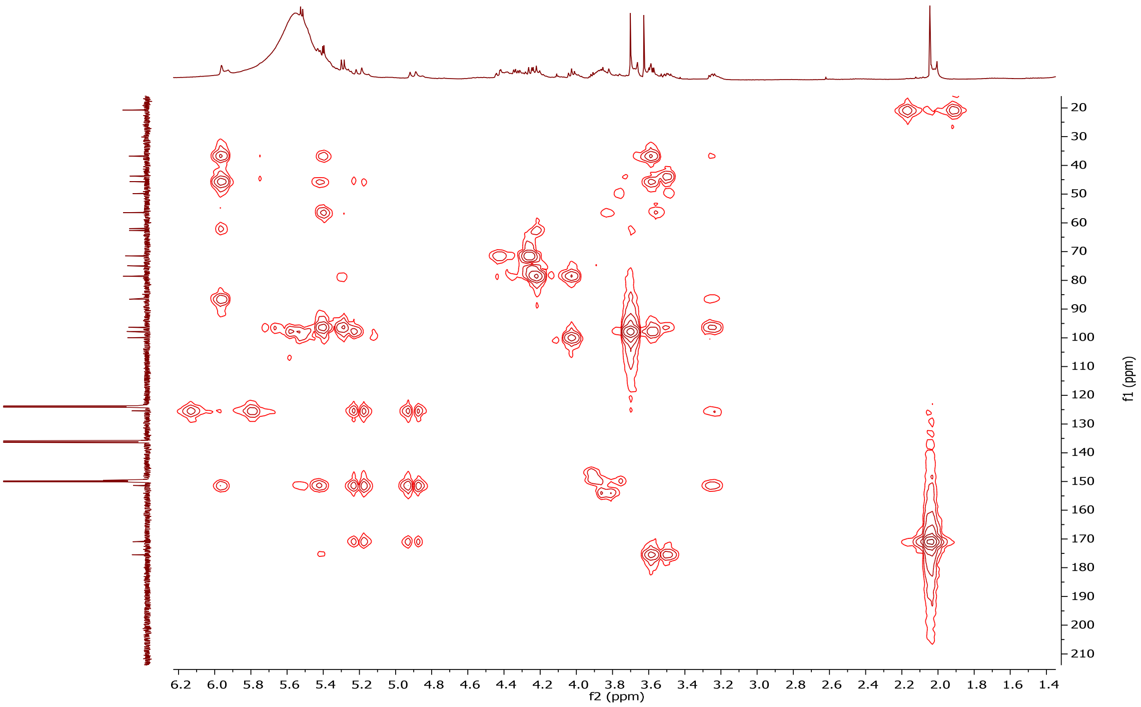

Figure S18. HMBC spectrum of **3** in pyridine-*d*5.

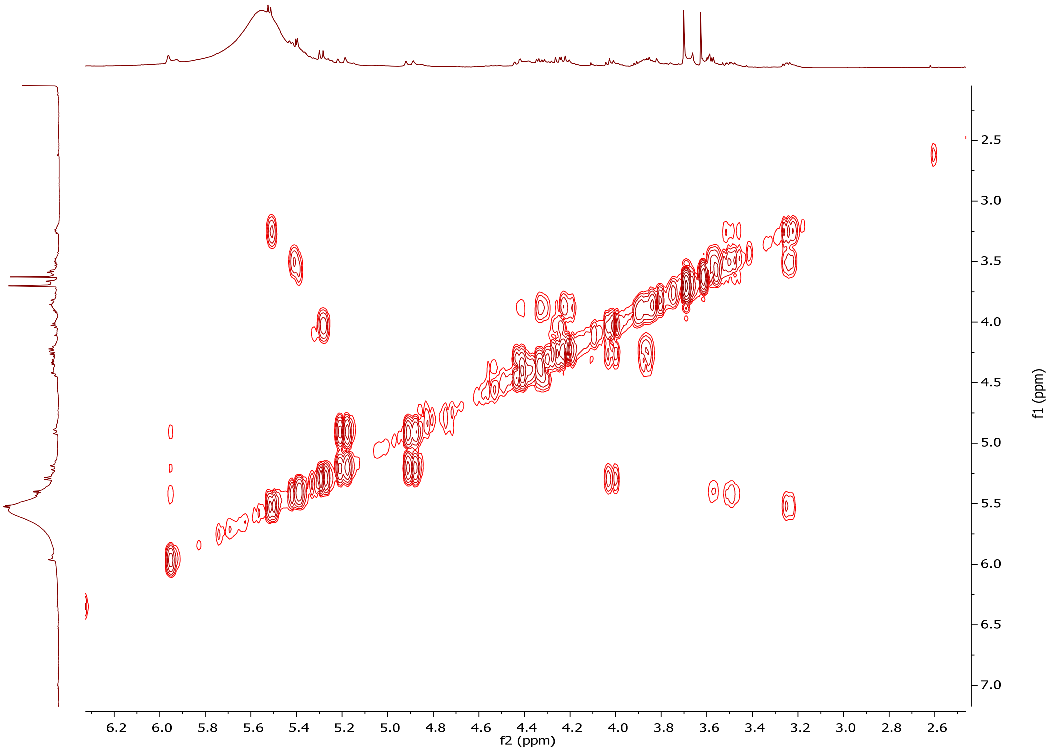

Figure S19. COSY spectrum of **3** in pyridine-*d*5.

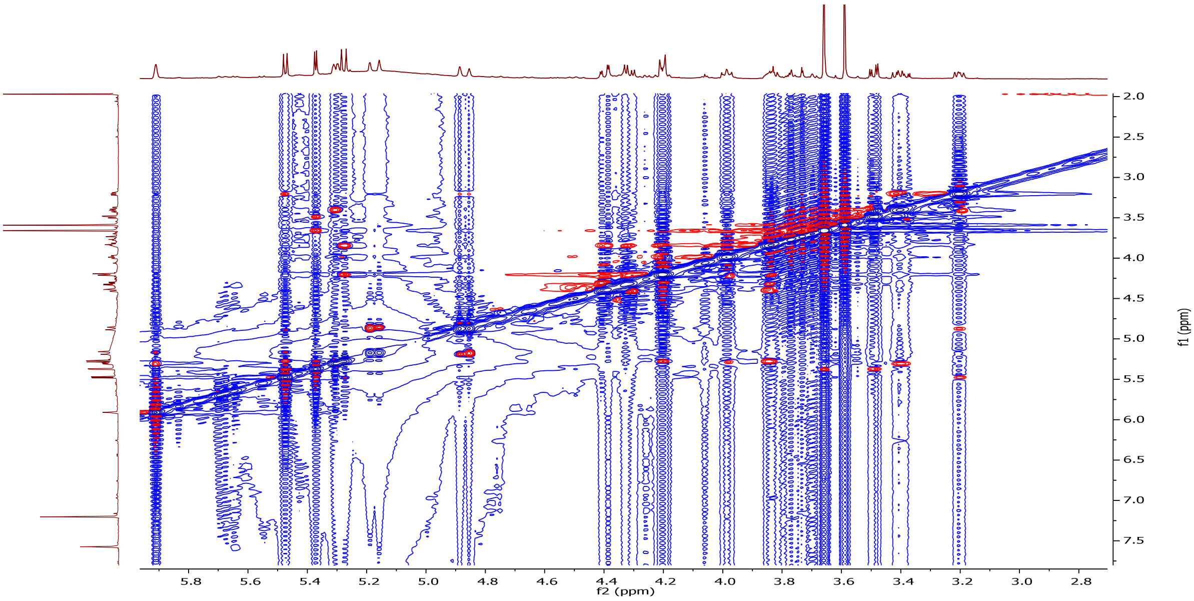

Figure S20. NOESY spectrum of **3** in pyridine-d_5.

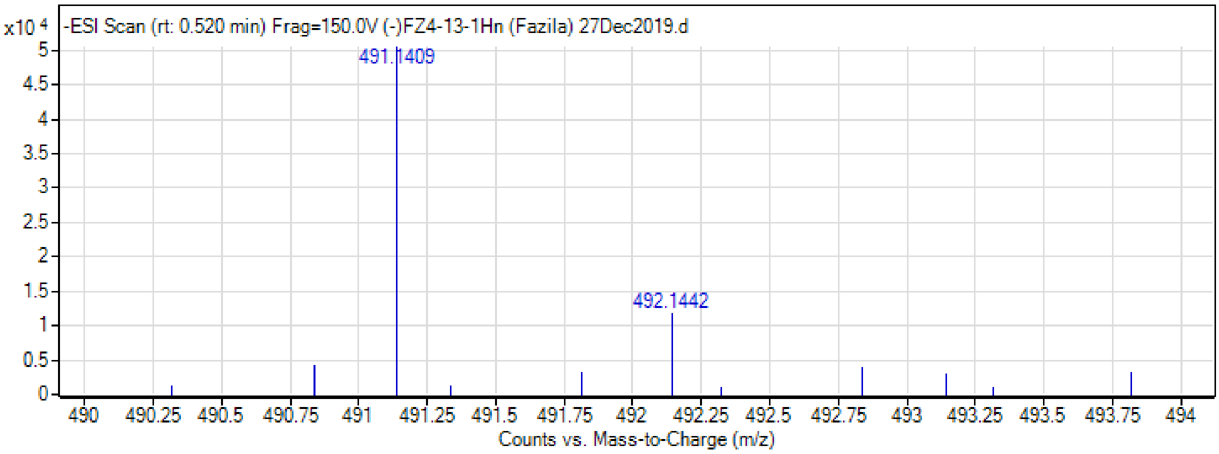

Figure S21. HRESIMS of **3**.

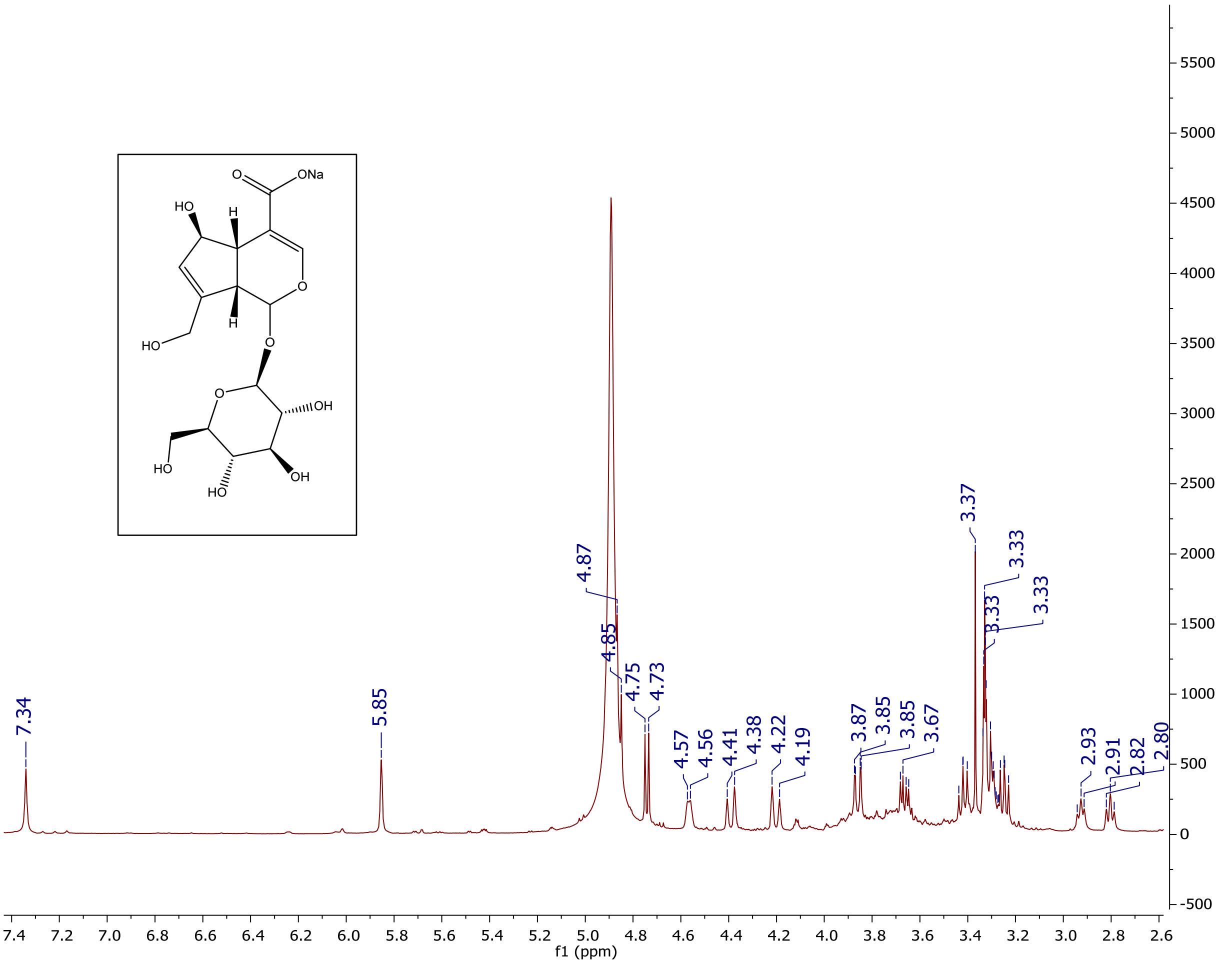

Figure S22. ¹HNMR spectrum of **4** in CD_3OD.

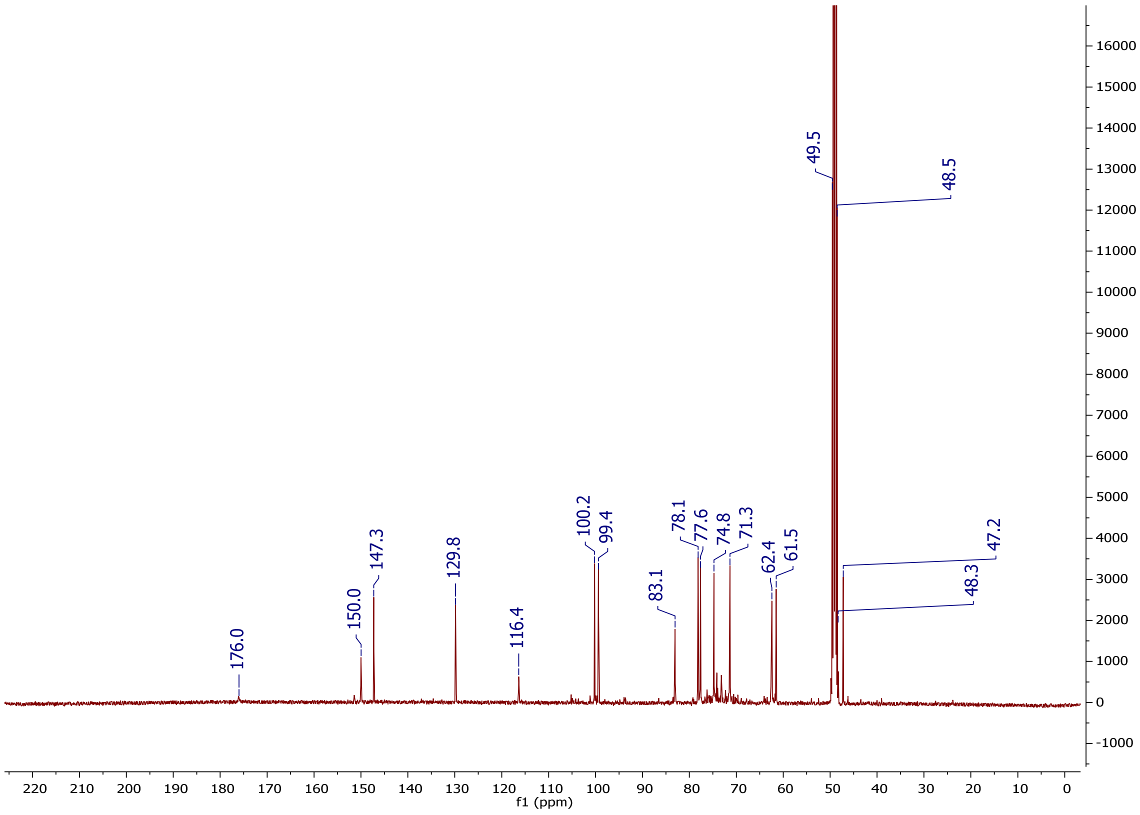

Figure S23. ¹³CNMR spectrum of **4** in CD_3OD.

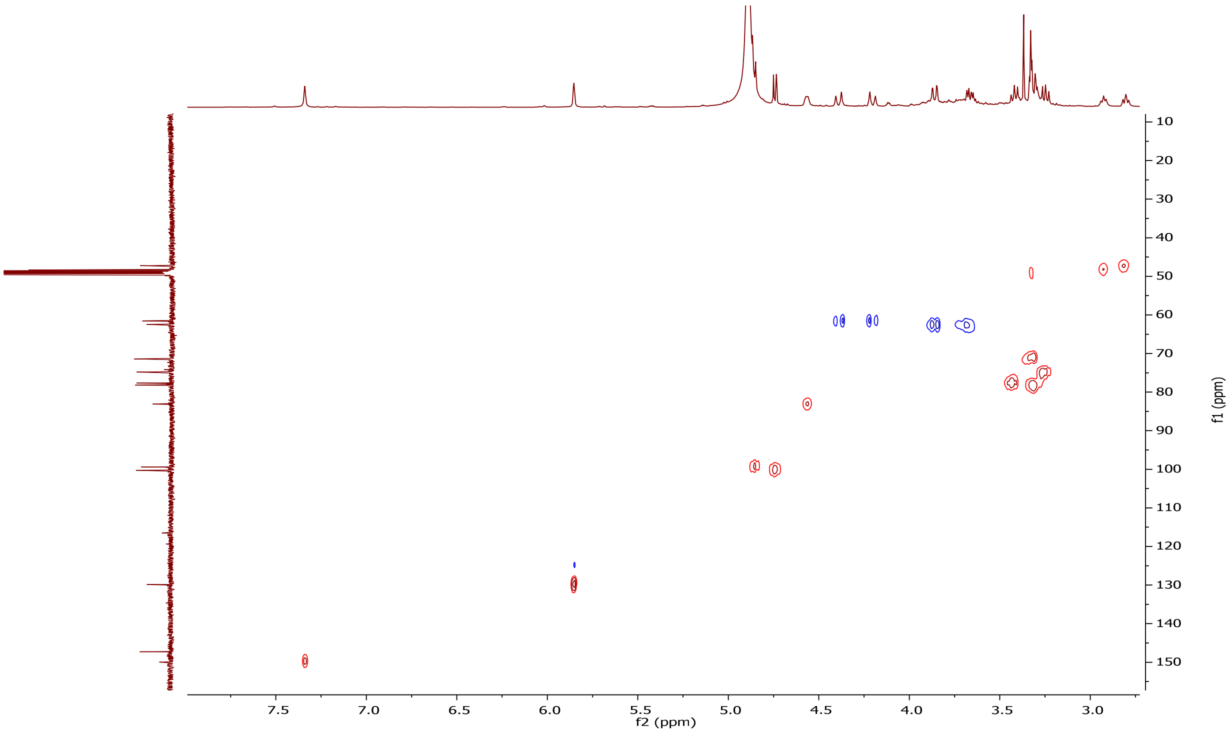

Figure S24. HSQC spectrum of **4** in CD_3OD.

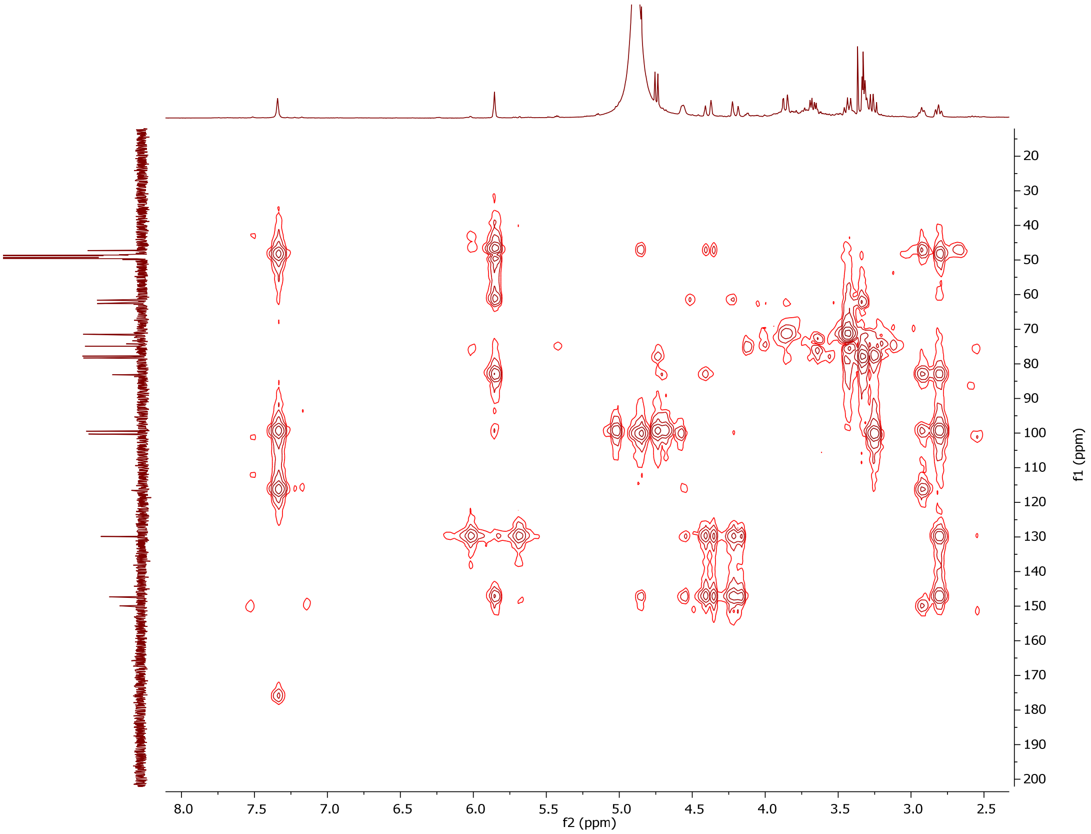

Figure S25. HMBC spectrum of **4** in CD_3OD.

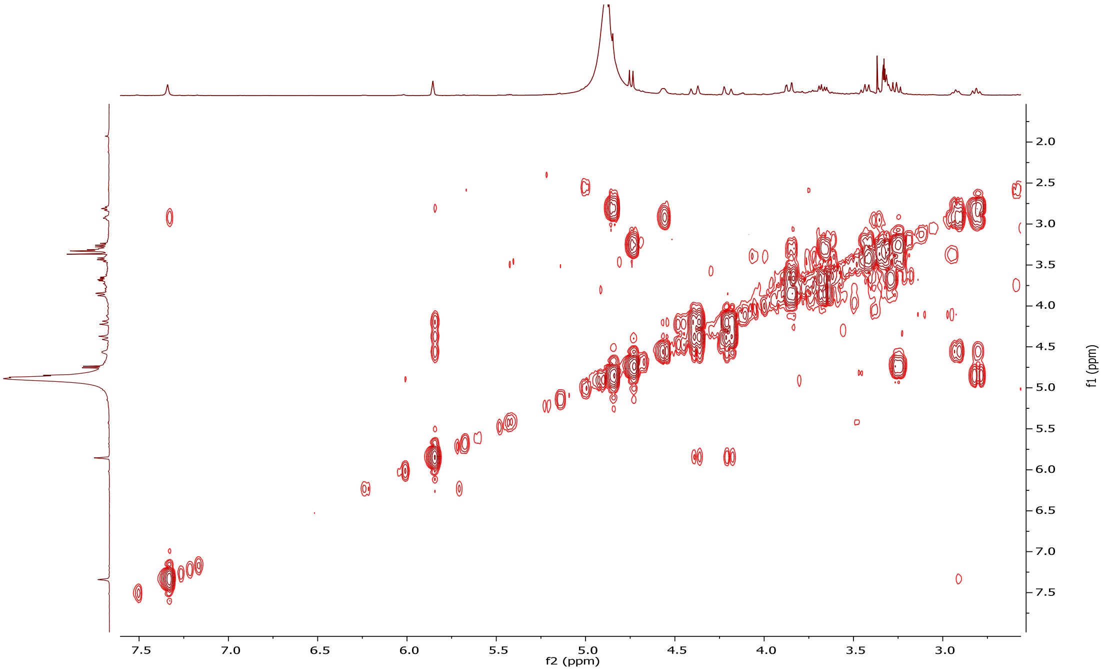

Figure S26. COSY spectrum of **4** in CD_3OD.

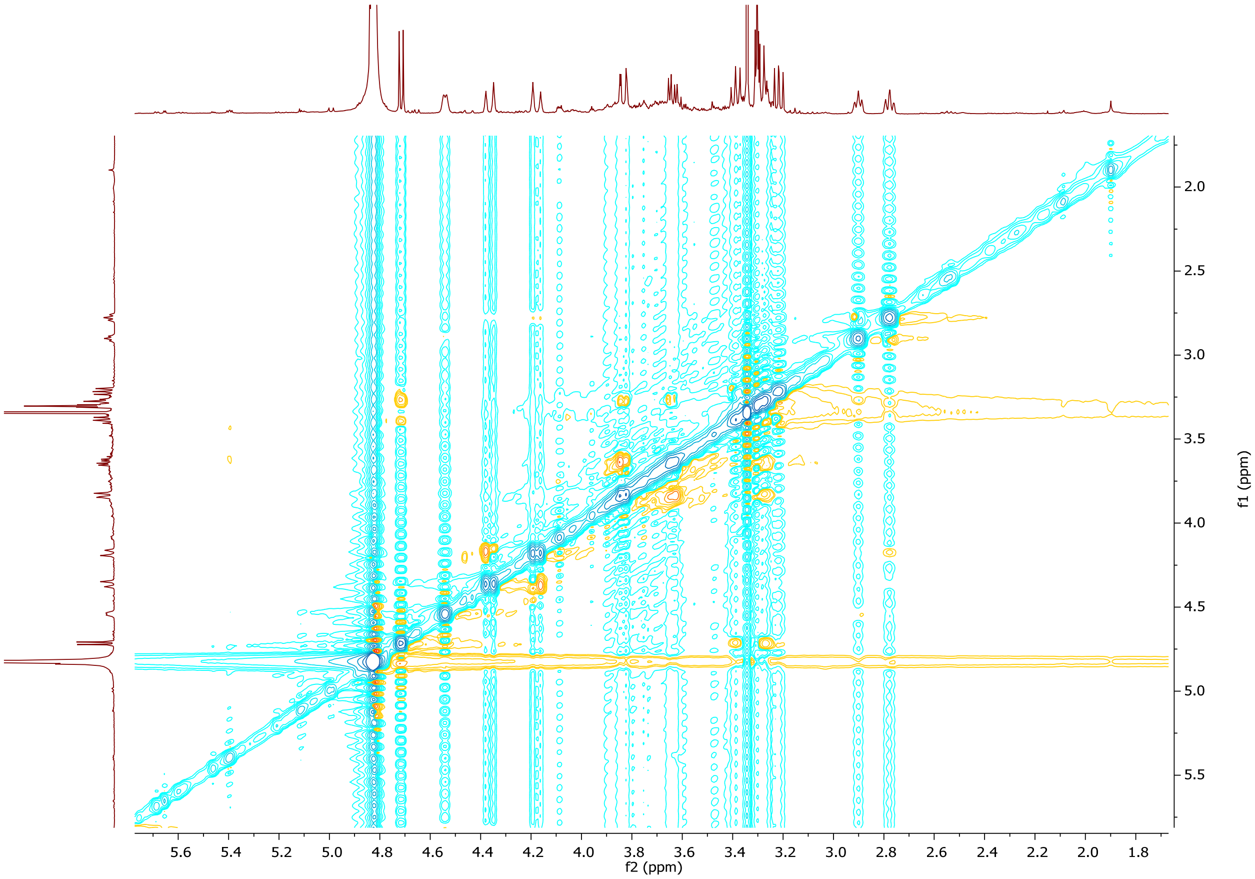

Figure S27. NOESY spectrum of **4** in CD_3OD.

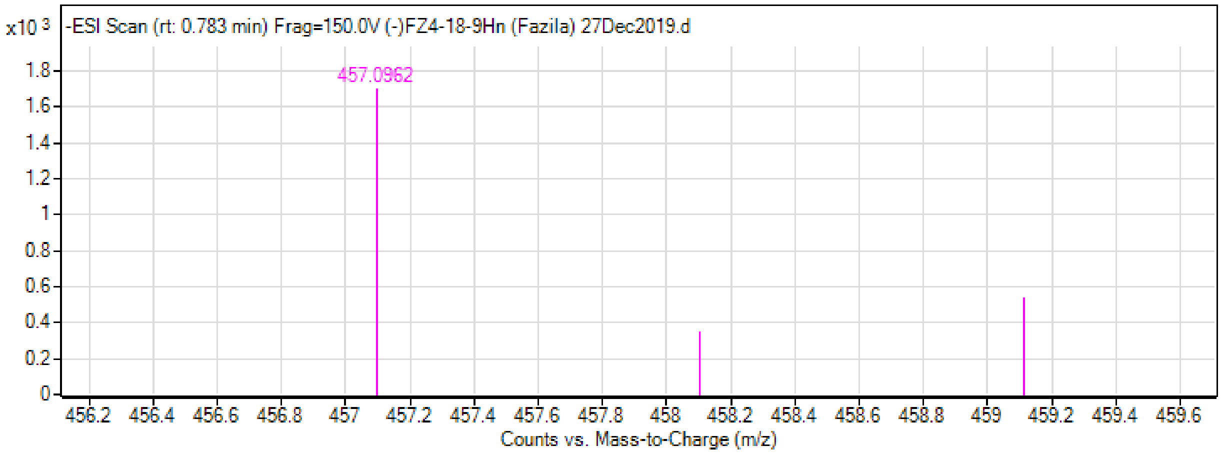

Figure S28. HRESIMS of **4**.
